# Mapping silver eel migration routes in the North Sea

**DOI:** 10.1038/s41598-021-04052-7

**Published:** 2022-01-10

**Authors:** Pieterjan Verhelst, Jan Reubens, Johan Coeck, Tom Moens, Janek Simon, Jeroen Van Wichelen, Håkan Westerberg, Klaus Wysujack, David Righton

**Affiliations:** 1 Marine Biology Research Group, Ghent University, Krijgslaan 281, 9000 Ghent, Belgium; 2 Research Institute for Nature and Forest (INBO), Aquatic Management, Havenlaan 88, bus 73, 1000 Brussels, Belgium; 3 Flanders Marine Institute (VLIZ), Wandelaarkaai 7, 8400 Ostend, Belgium; 4 Institute of Inland Fisheries E.V. Potsdam Sacrow, Im Königswald 2, 14469 Potsdam, Germany; 5 Institute of Freshwater Research, Swedish University of Agricultural Sciences (SLU), 178 93 Drottningholm, Sweden; 6 Thünen Institute of Fisheries Ecology, Herwigstraße 31, 27572 Bremerhaven, Germany; 7 Centre for Environment, Fisheries, and Aquaculture Science (Cefas), Pakefield Road, Lowestoft, NR33 0HT UK

**Keywords:** Animal migration, Behavioural ecology, Marine biology

## Abstract

Recent developments in tracking technology resulted in the mapping of various marine spawning migration routes of the European eel (*Anguilla anguilla*). However, migration routes in the North Sea have rarely been studied, despite many large European rivers and hence potential eel growing habitat discharge into the North Sea. In this study, we present the most comprehensive map to date with migration routes by silver European eels in the North Sea and document for the first time successful eel migration through the English Channel. Migration tracks were reconstructed for 42 eels tagged in Belgium and 12 in Germany. Additionally, some eels moved up north to exit the North Sea over the British Isles, confirming the existence of two different routes, even for eels exiting from a single river catchment. Furthermore, we observed a wide range in migration speeds (6.8–45.2 km day^−1^). We hypothesize that these are likely attributed to water currents, with eels migrating through the English Channel being significantly faster than eels migrating northward.

## Introduction

Anguillid eel populations face a dramatic global decline due to habitat loss, migration barriers, climate change, pollution, infection by non-native parasites and overexploitation^1^. The recent population decline to historical lows has resulted in the inclusion of many anguillid species on the IUCN Red List^2^; the European eel (*Anguilla anguilla* L.) has the highest status of concern (“critically endangered”) of all 19 anguillid (sub)species. Despite a substantial amount of studies and the efforts of researchers to clarify the various aspects of the life cycle of the species, a complete understanding of some aspects is still lacking, and there are many that still continue to be unclear^3^. Some aspects are well known: the European eel is a facultative catadromous fish found in coastal and freshwater systems from northern Africa to northern Europe while in the yellow eel (growth) stage; it subsequently undertakes a spawning migration into the Atlantic Ocean during the silver eel stage. The Sargasso Sea (ca. 20–30°N, 48–79°W)^4^ is traditionally assumed to be the spawning area of the European eel, but this assumption is largely based upon the discovery of the youngest larval stage (i.e. leptocephalus larvae) in the area early in the twentieth century^5^. However, more than a century after Schmidt’s discovery, spawning European eels nor eggs have ever been found in the ocean and large parts of their marine migration routes thus remain shrouded in mystery. This largely follows from a lack of suitable methods to track eels once they have reached the open ocean. Conventional mark-recapture tagging methods, for instance, are of limited use because eels are only rarely caught in oceanic waters.

Telemetry is a well-established technique to study the migration routes and behavioural ecology of animals in the wild. Over the past two decades, telemetry and tagging technology have benefitted substantially from miniaturization and software development^6,7^. Acoustic, radio and passive-integrated-transponder (PIT) telemetry depend on detection stations and are used to track eels in freshwater systems^8–11^. However, because radio and PIT signals are attenuated in saline waters, acoustic telemetry arrays are preferentially used in estuarine and marine environments^12–15^. Needless to say it is impossible to cover whole seas and oceans with acoustic arrays. Hence, other techniques are needed to track eels over large distances in the marine environment. Archival data loggers are undoubtedly the most suitable technology to date. These devices can be implanted in the eel’s abdominal cavity and store information on environmental variables like temperature and pressure (i.e. depth). The data loggers can be retrieved when an eel is caught or float to the surface by a floatation collar when the eel has died. More commonly, the data loggers are attached externally and a release mechanism is activated after a pre-programmed time. After floating to the surface, the devices either transmit the data to a server via the ARGOS satellite system (i.e. pop-off satellite archival transmitters (PSATs)), or they wash ashore and need to be retrieved and returned by the accidental finder (i.e. pop-off data storage tags (PDSTs)) so that the data can be downloaded. The use of archival data loggers has already led to the mapping of three different migration routes from Europe in the direction of the Sargasso Sea: a ‘Nordic’ route from Scandinavia to the north of the British Isles into the Atlantic, a second route from the west coast of Ireland towards the Azores, and finally one from the central Mediterranean through the Gibraltar Strait^16^.

However, the migration routes of eels leaving the North Sea remain largely unknown. This is a critical knowledge gap in any conservation strategy of European eel, because the North Sea is an important area for silver eels that migrate out of various large European rivers that function as important catchments for the yellow eel stage, such as the rivers Rhine (basin: 197,000 km^2^), Elbe (basin: 148,268 km^2^), Weser (basin: 46,259 km^2^), Glomma (basin: 41,965 km^2^) and Meuse (basin: 34,364 km^2^). Additionally, all eels leaving the Baltic Sea have to cross the North Sea to reach the Atlantic Ocean^12,17^. To date, only seven studies have reported on eel migration routes in the North Sea based on acoustic telemetry and archival data loggers. Five of these included only a limited number (≤ 14) of tracked eels and did not describe any eel exiting the basin^18–22^. Although these studies are limited, they have shown that at least a proportion of the European eels migrate south-westwards towards the English Channel to reach the Atlantic Ocean^18,20^, while others seem to migrate north^21^. The two remaining studies, based on a much larger number of individuals, reported a ‘Nordic’ route for eels leaving the Baltic along the Norwegian Trench over the British Isles^12,16^; this route is probably joined by the northward migrating individuals reported in the other studies. Since eels migrate along various routes, it can be assumed that eels leaving a specific catchment take the same route because they are subject to similar environmental conditions and start migration at a similar distance to the spawning site. Furthermore, different routes of varying lengths could show discrepancies in migration speed, so the eels would arrive together with conspecifics at their spawning grounds.

Here, we use archival tag data from a total of 54 silver eels released in Belgium and Germany to assess the direction and speed of migration to the Atlantic Ocean. Our hypothesis was that eels starting their migration from the same catchment would follow the same route.

## Results

### Tag and data retrieval

**Figure 1:**
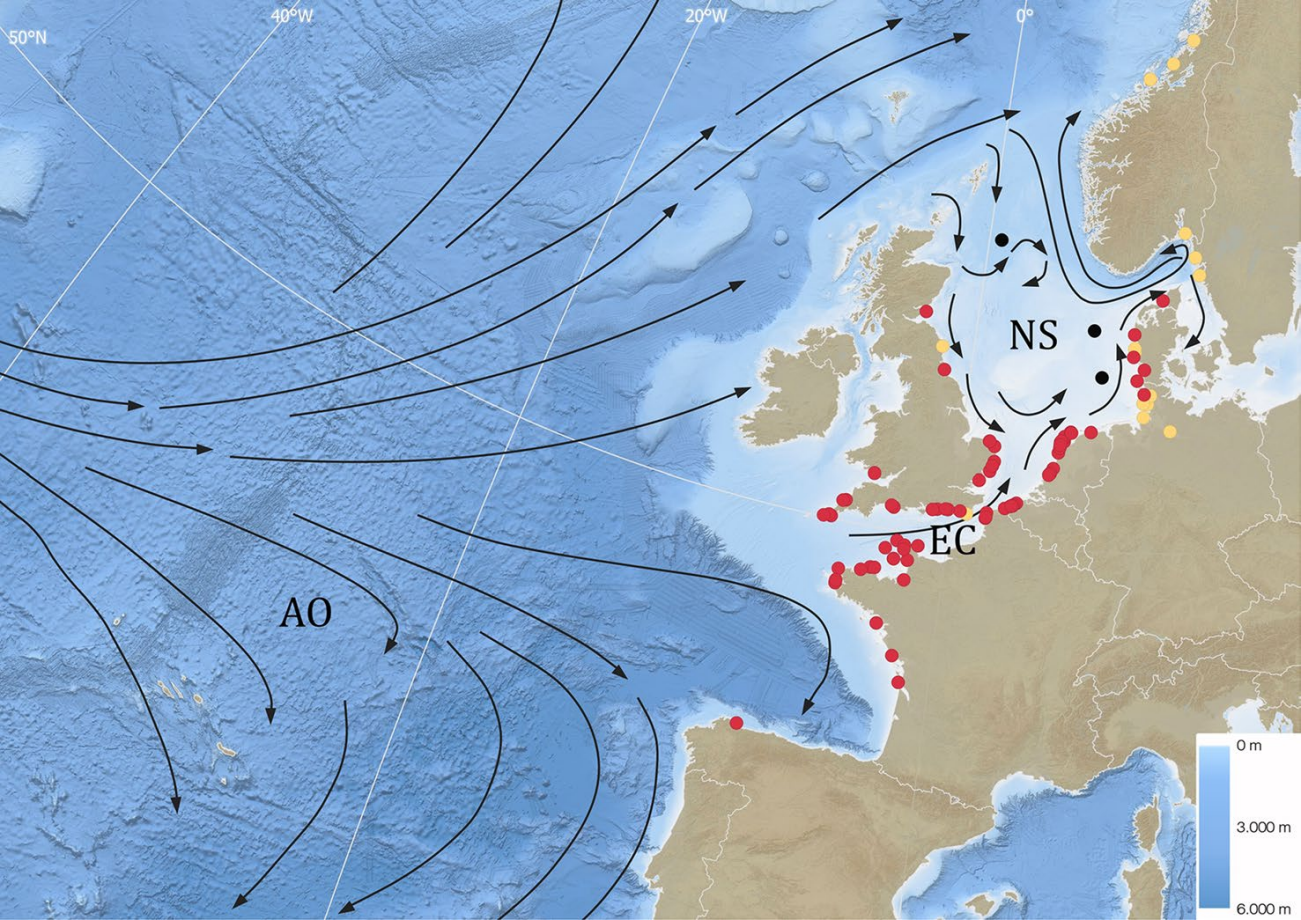
Bathymetric map of the North Sea (NS), English Channel (EC) and north-eastern part of the Atlantic Ocean (AO). The dominant surface currents are indicated with black arrows (figure based on reports from the European Environment Agency; https://www.eea.europa.eu/publications/report_2002_0524_154909). The retrieval positions of the PDSTs and pop-off positions of the three PSATs are indicated with dots (red: Belgian eels, yellow: German eels; black: PSAT pop-off positions). The legend of the bathymetry is indicated in the bottom right corner.

From the 320 tagged eels, datasets from 96 tags (30%) were retrieved (76 Belgian and 20 German eels) (Fig. 1, Table 1, Supplementary Table S1), which included data recovery from four PSATs. PDST retrieval of the Belgian eels (32%) was higher than that of the German eels (21%). Upon retrieval, the PDSTs were sent to the researchers together with the retrieval position and date, so the data could be downloaded from the devices. Four out of seven PSATs transmitted the data to the user via the ARGOS satellite system. However, one PSAT did not deliver a successful transmission, hence the pop-off position could not be identified, nor was the data useful.

**Table 1:** The number of tagged eels, retrieved tags and useful datasets with tracks ≥ 100 km per release location and period. The average length ± SD and weight ± SD is indicated.

Country	Release location	Period	Length (mm)	Weight (g)	Total tagged	Retrieved tags	Useful datasets
Belgium	Yser Estuary	2018	806 ± 43	1059 ± 169	102	29	9
		2019	837 ± 48	1226 ± 225	60	27	17
		2020	826 ± 60	1176 ± 231	76	20	16
Germany	River Eider	2011	896 ± 26	1488 ± 159	7	4*	3
	Elbe Estuary	2012	755 ± 38	839 ± 148	45	8	3
	River Eider	2012	795 ± 58	1065 ± 291	30	8	6

*PSATs attached to the eels in the River Eider in 2011 were not retrieved, but the data was obtained via transmission by the ARGOS satellite system.

### Tag fate

**Figure 2:**
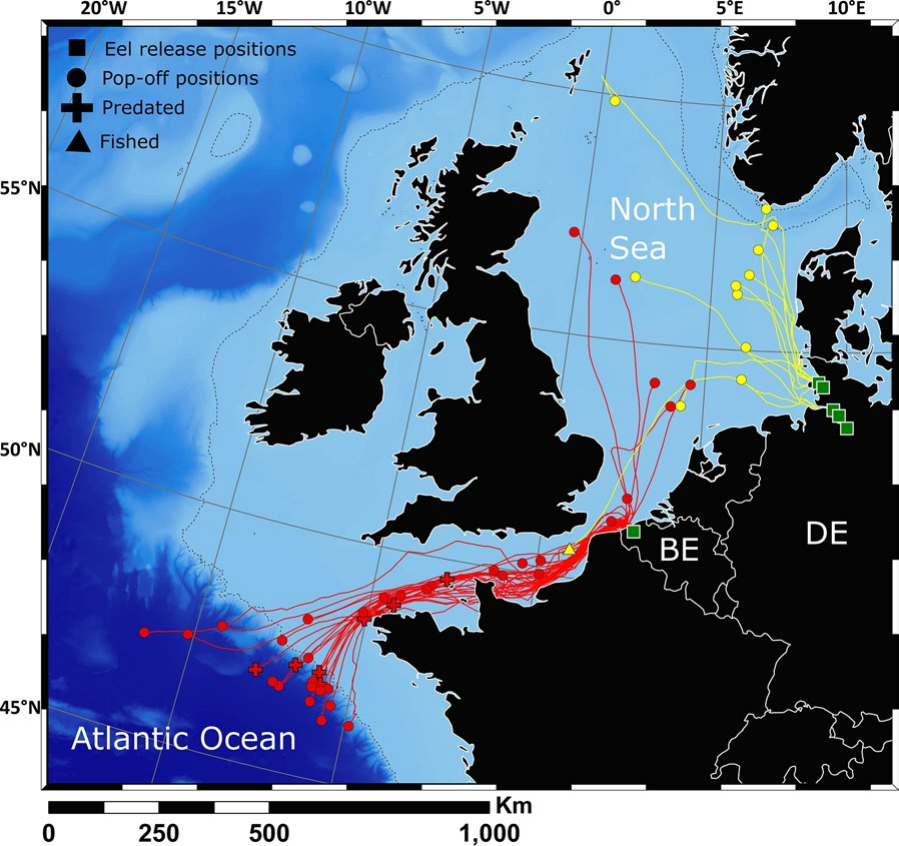
The reconstructed migration routes of the 42 Belgian (red) and 12 German eels (yellow). Predation is indicated by a cross and the fished eel in the English Channel by a triangle. Other premature and programmed pop-offs are shown with a circle. The eel release positions are indicated with a green square. The 200-m depth contour is delineated with a dotted line. The map was generated with ArcGIS^24^.

Apart from two German eels released in 2012, all tags surfaced before the programmed pop-off date; for 82 of these, the reason was unknown. Of the remaining 12 tags, 11 were predated and one was fished in the English Channel. Ten of the 11 predation events occurred on Belgian eels as they migrated out of the English Channel into the Atlantic Ocean (Fig. 2). The single German eel predation event occurred as the eel exited the Elbe Estuary. Note that this resulted in a track < 100 km, hence this data was excluded for trajectory reconstruction analysis. For four predation events, the predator was an endothermic fish because the temperature sensor registered values ca. 10 °C higher than the ambient water temperature (Supplemental Fig. S1), but below 30 °C. Another three cases showed predation by a marine mammal because the temperature rose to ca. 35 °C. In one of those cases, the predator was likely a pilot whale species (*Globicephala* sp.) given the frequent 20-min dives to > 400 m depth (Supplemental Fig. S2)^23^. The four other predators could not be identified, yet predation was likely because for one tag (A15777) the temperature rose briefly before the sensors broke down, while the other three showed highly atypical eel behaviour (tags A09355, A17521 and A09355).

### Migration routes

From the 96 retrieved tags, 54 contained reliable datasets with ≥ 100 km of net tracking distance in the marine environment (42 Belgian and 12 German datasets) (Table 1). The geolocation modelling revealed that both Belgian and German eels followed either a northern or southwestern migration route (Fig. 2). The majority of the Belgian eels migrated through the English Channel (n = 37, 88%), while only five (12%) moved northward. Of the German eels, ten migrated north (83%) and two eels (17%) headed southwestward towards the English Channel.

The average total tracking distance ± SD for the Belgian eels was 604 ± 267 km (range: 117–1145 km) during 27 ± 15 days (range: 4–68 days). They were all tagged and released between October and December. German eels were tracked on average over a distance of 360 ± 195 km (range: 196–759 km) for 32 ± 14 days (range: 15–68 days) and started between September and December (for information per eel, see Supplementary Table S2).

### Migration speed

The eel sizes did not differ significantly between Belgium and Germany (two-sample t-test, t(52) = 1.18, *p* = 0.24), excluding a size-bias on the results. The average migration speed ± SD for all 54 eels was 22.3 ± 9.9 km day^−1^ (range: 6.8–45.2 km day^−1^). Belgian eels (25.4 ± 8.7 km day^−1^ (range: 6.8–45.2 km day^−1^)) migrated twice as fast compared to German eels (11.5 ± 4.5 km day^−1^ (range: 7.2–21.5 km day^−1^)) (two-sample t-test, t(52) = 5.28, *p* = 2.57e^−6^). Since eels in both Belgium and Germany either migrated northward or south-westward, we compared migration speeds per route per country (i.e. south-westward Belgium, northward Belgium, south-westward Germany and northward Germany). However, there was not enough data for all routes (unbalanced design and relatively short tracks for the German eels) to get sound statistical results, hence we only report the different migration speeds that could help future studies. The southward migrating Belgian eels migrated on average 26.4 ± 8.4 km day^−1^ (range: 6.8–45.2 km day^−1^) and those migrating to the north 18.0 ± 8.4 km day^−1^ (range: 12.3–32.8 km day^−1^)). This was considerably faster compared to the German eels. German eels migrated southwestward at a speed of 14.4 ± 10.1 km day^−1^ (range: 6.8–21.5 km day^−1^), while those taking the northern route migrated at 10.9 ± 3.3 km day^−1^ (range: 7.4–16.5 km day^−1^).

The linear mixed effects model for the Belgian eels showed a significant positive relationship between daily migration speed and longitude, indicating eels slowed down as they moved westward and reached the ocean (Table 2). No significant effect was observed with latitude. The random effect ‘tag ID’ explained 38% of the variation in the model. In contrast, the model for the German eels revealed that the migration speed significantly decreased with higher latitude, while no effect of longitude was found. In this model, ‘tag ID’ described 23% of the variation.

**Table 2:** The output of the linear mixed effects models for the Belgian and German eels with the estimated value for the intercept and covariates, the standard error (SE), degrees of freedom (DF), t-value and *p*-value.

Eel group	Explanatory variables	Value	SE	DF	t-value	*p*-value
Belgian eels	Intercept	42.87	23.95	1121	1.79	0.07
	Longitude	0.72	0.20	1121	3.55	< 0.01
	Latitude	− 0.37	0.47	1121	− 0.77	0.44
German eels	Intercept	125.21	24.44	384	5.12	< 0.01
	Longitude	0.02	0.26	384	0.07	0.95
	Latitude	− 2.06	0.42	384	− 4.86	< 0.01

## Discussion

This study is the first to report on the migration of silver European eels from the North Sea into the Atlantic Ocean through the English Channel (i.e. the Channel route). In addition to this southwestern route, our results also indicate that a small but significant fraction of silver eels leave the North Sea via a Nordic route to the north of the British Isles, where they are likely to join the main migration route of Baltic Sea eels^12^. Caution is due when comparing results from the Belgian and German eels, because they were tagged over different time periods (i.e. 2018–2020 and 2011–2012, respectively), and because the PSAT-tagged German eels were kept in the laboratory for several weeks prior to their release. Our data do not allow to address the former issue. More generally, despite the substantial number of eels for which migration data were included in the present study, our data set was still too limited and unbalanced to analyse the impact of the different release periods (not just years, but also months) or the various release locations. With respect to the possible effect of the holding period on eel migration behaviour, we know of no studies which have reported such effects.

The basis upon which eels choose either route remains unknown. Since the European eel is considered panmictic under the assumptions of random mating and random larval dispersal^25^, a genetic basis for divergent route choices is unlikely. An alternative explanation is that leptocephalus larvae and glass eels have their migration route “imprinted” during their migration from the Atlantic to their nursery grounds, since these juvenile eels are transported by the North Atlantic Current and can enter the North Sea via both the English Channel and along the north coast of Scotland^26,27^. The imprinting hypothesis has received some support from mark-recapture studies in Scandinavia, where silver eels originating from stocked glass eels missed the outlet of the Baltic Sea^28^. However, evidence is far from conclusive; as an example, another mark-recapture study concluded that both naturally recruited and stocked eels originating from France and the UK migrated from Sweden towards the outlet of the Baltic Sea^17^. In addition, Westerberg et al.^12^ observed that naturally recruited and restocked eels both took the northern migration route from Sweden to the Atlantic Ocean. Similarly, stocked American eels (*A. rostrata* Lesueur) were able to leave the St. Lawrence River along the same route as their naturally recruited conspecifics^29^. This indicates that silver eels do not solely rely on an imprinted route and are potentially guided by specific orientation cues. Note that in the present study, we could not determine whether the tagged eels were natural recruits or resulted from stocking, because eels from France and the UK have been stocked in both study areas in Belgium and Germany in at least the past 15 years^30^.

Currents are one potential trigger that could play an important role in eel orientation and route choice. It is widely accepted that silver eels are triggered and guided by an increased water discharge in freshwater systems to migrate seaward^11,31,32^ and also orient along tidal currents in estuaries^13^. Because silver eels respond to specific water flows, it is likely that they also use currents at sea for orientation. The North Sea is a dynamic area in terms of currents with tidal, wind-driven, topographic and baroclinic effects all affecting prevailing current directions and strengths^33^. The strong currents running through the narrow English Channel potentially trigger the majority of the silver eels from Belgium to take that route. The majority of the German silver eels probably migrate northward as they follow the predominantly northern currents running along the western Danish coast into the deep Norwegian Trench (Fig. 1).

There are several other environmental cues which may affect eel orientation^34^, irrespective of whether they are imprinted or not. The observation that anosmic silver eels are substantially disoriented in estuaries, for instance, suggests that olfactory cues may contribute to seaward orientation^35,36^. Magnetism^37^ and environmental sound^38^ are but two possible factors which may affect eel orientation during migration.

The migration route choice likely has important consequences for the success of both migration and reproduction. The Nordic route is substantially longer than the Channel route for eels coming from the Southern Bight of the North Sea (ca. 1000 km longer to the Azores, the furthest location European eels have ever been tracked^16^). Consequently, the Nordic route will require a higher energy expenditure on swimming and, because silver eels do not feed during migration^39^, a longer journey will likely leave less energy for gonad development and spawning. This is especially significant because anguillid eels are semelparous species, and therefore only have one chance in their life to reproduce^40^. However, we cannot exclude the possibility that eels follow specific currents which compensate for the increased bio-energetic cost of a longer journey, nor that trade-offs exist which balance the extra fitness cost of such a longer migration route.

One such trade-off might be a differential predation risk depending on the chosen migration route. Silver eel are particularly vulnerable to predation in shallow marine habitats such as the outlet of estuaries, the continental shelf^16,41–43^ or a narrow sea passage like the Gibraltar Strait^44^. Although we could only confidently assign 11 among the 96 retrieved tags to predation, all the other tags detached prematurely due to unknown causes, so the prevalence of predation may well have been severely underestimated. Among the 11 certain predation events, most occurred on the continental shelf as eels were leaving the English Channel, supporting the idea that narrow sea corridors and estuary outlets are potential predation hot spots. The German eels, by contrast, may experience a lower predation risk in the deep waters of the Norwegian Trench, potentially balancing the higher bioenergetics cost of the longer Nordic route by a higher chance of survival. In this context, it is important to note that externally attached PDSTs and PSATs can increase predation susceptibility due to more prominent water vibrations, which can be picked up by the sensory system of predators, or a higher visibility^45^.

Although the migration speeds can differ between studies depending on the geolocation method used, our results (range: 6.8–45.2 km day^−1^) confirm the broad migration speed spectrum exhibited by silver European eels at sea according to previous studies (range: 3–47 km day^−1^)^12,16,18^. This broad range is at the basis of the idea of a “mixed migratory strategy”, where a proportion of the migrating eel population may reach the spawning location within the spawning season that follows their migration onset, while others will only arrive a year later^16^.

Nonetheless, with an average speed of 25.4 km day^−1^, the Belgian eels migrated over twice as fast than the German eels (11.5 km day^−1^). This pattern was also observed when comparing the speeds between the routes, with Belgian eels following the Nordic route migrating at 18 km day^−1^ and the German eels at 10.9 km day^−1^. Belgian eels taking the Channel route migrated at 26 km day^−1^, while German eels migrated at 14 km day^−1^. This coincides with a tendency of eels taking the Nordic route to migrate at a slower pace than their conspecifics which take the Channel route. Additionally, silver eels slowed down as they progressed westward or northward. This may well be linked to the prevailing currents, such as tidal currents. We hypothesize that eels apply selective tidal stream transport to migrate in an energetically efficient way, and migrate in the direction of the Atlantic Ocean during the ebbing tide while residing stationary on or in the bottom during the opposite tide^46^. Since eels apply this behaviour in estuaries^13,47^, it is reasonable to assume that they continue doing so at sea. The Belgian eels may keep a relatively fast speed as their release position is close to the narrow Strait of Dover, where the tidal currents through the English Channel are the strongest^33^. As their migration progresses, their migration speed declines as the current slows down towards the Atlantic Ocean. The current strengths in the North Sea are considerably lower than in the English Channel and decline from south to north^33^. Hence, if the migration speed of the German eels depends on current strength, this could explain why they slow down as well and migrate more slowly altogether. Although the current strength slightly increases from Germany towards the English Channel, it remains relatively constant along the coast of the Southern Bight, so German eels taking the south-western route may not increase their speed significantly. Moreover, along that route eels may not apply selective tidal stream transport due to the lower tidal strength, leading to slower speeds, as also observed for plaice (*Pleuronectes platessa* L.)^48^.

Although our results illustrate that PDSTs represent a valuable technology to track European eel during its migration at sea, the success rate of obtaining valuable datasets is lower compared to that of PSATs (64–100%)^49^. The main reason for this difference is obviously that PDSTs need to be found and retrieved. However, applying PDSTs in areas with touristy beaches and strong prevailing currents directed towards the coast can enhance the retrieval rate. An obvious advantage of PDSTs over PSATs are the very detailed, high-resolution datasets stored by the former. Another advantage of the PDSTs is that they are smaller than the PSATs (ca. one third in weight and half the length). While laboratory studies on the impact of external PSAT-tagging on eels have observed no significant effect on the eel’s optimal swimming speed^50–52^ (note that the three German eels with PSATs in our study also did not have a lower migration speed than the German eels tagged with PDSTs, despite the considerably smaller size and weight of the latter), the cost of transport did increase significantly by at least 26%^50,52^.

To conclude, this study shows that eels taking different routes can have highly variable migration speeds and therefore adds to the “mixed migratory strategy”^16^. The fact that eels can take both routes, partly independent of their catch and release location, illustrates the flexible life strategy of the species. Mapping the migration routes in the North Sea builds upon the fundamental knowledge on eel migration and the potential hazards they encounter (e.g. predation). This information is crucial to restore the population^53^. Despite the new insights, it stresses the various knowledge gaps in our understanding of how eel migration, but also fish migration in general, works, such as orientation mechanisms and migration speed ranges in relation to spawning timing. Such knowledge is essential to evaluate if, for instance, translocated eels can find their way to the spawning grounds at a similar speed as their non-translocated conspecifics do. This is highly relevant for the restocking programs in which anguillid eels are translocated from areas with high abundance to depleted catchments^12^.

## Methods

### Study area

The North Sea is a continental sea connected to the Atlantic Ocean through the English Channel in the southwest and between northern Shetland along the 61° latitude parallel to Norway in the north (Fig. 1). It is bordered by Norway, Denmark, Germany, the Netherlands, Belgium, France and the UK, and has a surface of 570,000 km^2^. The North Sea has an average depth of 95 m, yet maximum depths of ca. 700 m are found in the Norwegian Trench. The maximum tidal amplitude of the North Sea can reach up to 8 m, average winter sea surface temperatures are ca. 6 °C and average summer temperatures reach ca. 17°C^33^. The English Channel encompasses the marine strait between the UK and France. It covers 75,000 km^2^, has an average depth of 63 m, a maximum depth of 174 m and can reach a maximum tidal amplitude up to 12 m. The average winter and summer sea surface temperatures in the English Channel are ca. 5 and 20 °C, respectively^54^.

### Tagging

In total, 320 silver eels were tagged with pop-off archival tags (Table 1; Supplementary Table S2). In Belgium, 238 eels were caught and tagged at a drainage system upstream of the Yser Estuary (hereafter referred to as the Belgian eels) in 2018–2020 via nets that were attached to gravitational discharge sluice gates (coordinates: 51.127 N, 2.761 E) in October, November and December (n_2018_ = 102, n_2019_ = 60 and n_2020_ = 76). In Germany, 82 eels were tagged in 2011 and 2012. In early December 2011, seven eels were caught at Lake Plön (coordinates: 54.137 N, 10.334 E) with fyke nets. During September, October and November 2012, eels were caught in the Rivers Eider (n = 30; coordinates: 54.190 N, 9.093 E) and Havel (n = 45; coordinates: 52.419 N, 12.571 E) with fyke and stow nets, respectively.

Upon capture, the eels were anaesthetized with 0.3 ml/L clove oil (Belgium), 0.4 ml/L ethylene glycol monophenyl ether (Germany 2011) or 120 mg/L MS-222 (Germany 2012), and various morphometric characteristics were measured to identify the life stage^55^: total length (to the nearest mm), weight (to the nearest g), horizontal and vertical eye diameter (to the nearest 0.01 mm in Belgium and to the nearest 0.1 mm in Germany) and pectoral fin length (to the nearest 0.01 mm and 0.1 mm in Belgium and Germany, respectively). Given that their total body length was > 450 mm, all eels were considered female^55^. According to the morphometrics, five Belgian eels could be considered in the premigratory stage (FIII); however, based on visual inspection, they were considered silver eels (i.e. silver-coloured abdomen, dark grey on the dorsal side, jaw hinge not proceeding beyond the eye, enlarged eyes and dark coloured pectoral fins). The other 315 eels identified as silver eels based on both morphometry and visual inspection (201 FIV stage and 114 FV stage).

Eels weighing ≥ 550 g were externally fitted with a G5 PDST (CEFAS Technology Ltd, UK), which log temperature and pressure (providing information on depth). They were attached applying the three-point Westerberg attachment method^56^. Two tag types were used: one with a separate tag and pop-off mechanism (Germany) and one where both mechanisms were integrated (Belgium). The flotation collar of the PDSTs was painted bright red, contained contact information and a cash reward to stimulate retrieval by the general public (e.g. beach combers and fishermen). The seven eels caught in 2011 in Germany (minimum 1220 g) were fitted with PSATs (X-Tag, Microwave Telemetry Inc., USA), also using the Westerberg-method^56^. Like the PDSTs, the PSATs record temperature and pressure. After release, they drift to the surface and transmit the data to the user via the ARGOS satellite system (www.argos-system.org). For the specifications of the different tags, we refer to Supplementary Table S3.

Upon recovery from the anaesthetic, eels tagged with PDSTs were released close to their capture locations in the rivers Eider (coordinates 2011: 54.381 N, 9.009 E; coordinates 2012: 54.379 N, 9.013 E), Elbe (coordinates 1: 53.793 N, 9.402 E; coordinates 2: 53.569 N, 9.700 E; coordinates 3: 53.396 N, 10.171 E) and Yser (coordinates: 51.135 N, 2.757 E) (Table 1). The seven eels captured for PSAT tagging in 2011 were held for several weeks in the Thünen Institute of Fisheries Ecology, then tagged and released the same day; others were tagged in the field.

### Preprocessing

Once downloaded, the temperature and pressure data obtained from the PDSTs was subsampled to 1-min (Belgian eels) or 2-min (German eels) intervals to reduce the datasets and improve geolocation calculation time; this discrepancy is due to the minimum logging rate of the tags (Supplementary Table S3). Linear regression was applied to correct for pressure sensor drift over time. Indeed, pressure values increased over time even if the tag was kept at atmospheric pressure level. The regression was applied between 15 min before release and the moment the tag popped off and reached the surface, since the tag was then considered at sea level and hence to be under zero pressure.

The PSAT data were retrieved through the ARGOS satellite system as a subset with 15-min intervals and converted to values of pressure and temperature. Contemporaneous values of temperature and depth were not always transmitted due to the transmission method. As a consequence of the tag release programming, the transmission of the first position for one of the tags was only received five days after the tag reached the sea surface.

### Geolocation

The daily movements of each electronically tagged European eel were reconstructed using an adapted version of the tidal geolocation model of Pedersen et al.^57^. The geolocation model uses a novel Fokker–Planck based method to combine the tidal location method of Metcalfe and Arnold^58^ with a hidden Markov model (HMM), such that an individual’s daily location *d* is modelled conditionally on its previous location (*d* − 1), its inferred behavioural state *ds*, where behaviour is defined by a single diffusivity parameter (i.e. the maximum amount of movement permitted in a given day), and the observations made between *d* and *d* − 1. In this case, observations consisted of the recorded depth (m; D1, …, Dn) and temperature (°C; T1, …, Tn), where n is the number of measurements made per day (the HMM down-samples to 10-min intervals, hence 144 measurements per day), and any hydrostatic (tidal) data which are derived from the sinusoidal pressure cycle recorded in the depth data when a fish is at rest on the seafloor. In addition to bathymetry and tidal amplitude with phase, the model was developed to include sea surface temperature (SST), which can provide additional validation when fish are swimming at or near the surface (i.e. depth ≤ 20 m)^59,60^, and temperature at depth, which can provide additional validation when fish remain at depths well below the sea surface^61,62^.

The model was run in three different configurations for each recovered dataset: (i) using the tidal location model only (as for Pedersen et al.^57^), hereafter termed TLM geolocation; (ii) using the TLM plus sea surface temperature (as for Wright et al.^60^), hereafter termed SST geolocation, and (iii) using temperature at the surface and sub-surface, hereafter termed 3D geolocation (Supplemental Fig. S3). The final trajectory output for the PDST Belgian eels and PSAT German eels was obtained via 3D geolocation, while SST geolocation was used for the PDST German eels. The reason for this discrepancy is that the German PDST eels stayed closer to the coast and in shallower water. Consequently, the 3D geolocation results were more prone to error due to coastal influences on water temperature. As a result, we used the SST geolocation method for these datasets to obtain more reliable results.

Data for the model were derived from publicly available resources. Gridded global bathymetry data were obtained from the general bathymetric chart of the oceans (Gebco; British Oceanographic Data Centre, Liverpool, United Kingdom, 2009). Tidal constituents were obtained from the Oregon State University Tidal Prediction model, as described in Egbert and Erofeeva^63^. Sea surface temperature data were sourced from OSTIA^64^, while temperature at depth data were sourced from the operational Mercator global ocean analysis and forecast system^65^. These datasets were downloaded from the Copernicus Marine Environmental Model Service (CMEMS: documented here http://resources.marine.copernicus.eu/documents/PUM/CMEMS-GLO-PUM-001-024.pdf). Data were sourced so as to fit the spatial scale of the model (30°N to 80°N and from 110°W to 60°E) and coarsened to reduce model run-time by modifying the spatial grid to a 1/10^th^ of a degree resolution. The output of the model is a nonparametric probability distribution of the geographical position from which a most probable location, for each day at liberty, and a most probable movement path can be estimated.

Prior to running the model, a number of constraints and input parameters were defined to ensure that the model ran effectively. The recapture information was either set as (a) the latitude and longitude where the tag was recaptured, with a high confidence (< 5 km error) or (b) as an estimate of the location closest to that which matched the maximum depth and sea surface temperature on the day before the tag came to the surface, or the day before it was predated (low confidence: 50 km error). Model diffusivity (a proxy of the swimming speed of the fish) was set to low default values that matched the expected travelling speed of eels (ca. 20 km per day, maximum 50 km per day^16^). Two values were used; a low diffusivity value of 30 km d^−2^, corresponding to days on which vertical movement was low and infrequent, and a larger value of 100 km d^−2^, corresponding to days when vertical movement was high and frequent. These values broadly correspond to localized (resident) and migratory behaviours, respectively^57^. Finally, daily estimates of longitude were used to modify the weighting given to the likelihood areas generated from the SST-geolocation and the 3D geolocation. For eels that did not reach oceanic depths (i.e. depths > 200 m) and hence did not exhibit diel vertical migrations, the input estimates of longitude were based on a simple linear interpolation from release to estimated pop-up. However, for eels that did reach oceanic depths, the time of local noon was estimated (based on the timing of significant diel vertical migrations, as for Righton et al.^16^), and used to estimate longitude. Geolocation was conducted with MATLAB software^66^.

### Migration routes

Only datasets containing ≥ 100 km of net tracking distance were included for further analysis, leading to 54 datasets from the 96 retrieved tags and 320 tagged eels. The net tracking distance was identified as the distance along the reconstructed trajectory between the release of the tagged eel and the pop-off event. When an eel was ingested by a predator, leading to the tag tracking the predator rather than the eel, the data were excluded from the day the eel was predated. The 100 km cut-off point was arbitrarily chosen to select migration paths of sufficient length for further analysis (e.g. migration direction); tracks had a minimum deployment duration of 4 days.

### Migration speed

To exclude a size-effect, we first applied an independent two-sample t-test to confirm eel sizes (i.e. weight) did not differ between Belgian and German eels. The assumptions of normality (Shapiro–Wilk test), homogeneity of variances (F-test) and independence were met (weight measurements are individual-specific and therefore independent).

Next, an independent two-sample t-test was conducted to test if the total migration speeds (i.e. the ground speed along the reconstructed trajectory between the release of the tagged eel and the pop-off or predation event) differed between Belgian and German eels. The assumptions were tested and met as described above.

Finally, we tested if the daily migration speed (i.e. the ground speed along the reconstructed trajectory per day) differed according to the eel’s position (i.e. modelled latitude and longitude) via a linear mixed effects model. The tag IDs were implemented as a random effect to account for autocorrelation. Since the two-sample t-test showed a significant difference between Belgian and German total migration speeds, we performed a separate analysis on eels from both countries. Assumptions of normality, homogeneity of variances and independence were tested and met.

The migration speed analyses were conducted in R (version 3.6.3)^67^. The packages ‘lme4’ and ‘nlme’ were used to conduct the linear mixed effects model.

### Ethical statement

Eels were tagged using approved protocols by trained and individually licensed scientists working under national project authority in accordance with institutional and national guides for the care and use of laboratory animals. These guidelines are consistent with Institutional Review Board/Institutional Animal Care and Use Committee guidelines. Tagging in Belgium was carried out in accordance with the Belgian national and regional regulations for animal welfare and treatment (Permit ID: EC INBO-011). Tagging in Germany followed German legislation concerning care and use of laboratory animals, and ethical permission for the experiments was given by the Ministry of Energy, Agriculture, the Environment, and Rural Areas of the federal state Schleswig–Holstein (reference numbers V312-72241.123-34 (90-8/11) and V311-7224.123.3 (93-6/12) for tagging in 2011 and 2012 respectively).

## Supplementary Information

Supplementary Information.

## Data Availability

The data will be made available upon publication on the European Tracking Network database (https://www.lifewatch.be/etn/) and are available from the corresponding author on request.
