# Supplementary material for: Mapping silver eel migration routes in the North Sea
Source: Sci Rep. 2022 Jan 10;12:318. doi: 10.1038/s41598-021-04052-7 (PMC8748739; doi:10.1038/s41598-021-04052-7)
Supplement: Supplementary file 1 — Supplementary Information. [file 41598_2021_4052_MOESM1_ESM.pdf]

Mapping silver eel migration routes in the North Sea

Pieterjan Verhelst[1,2]*, Jan Reubens[3], Johan Coeck[2], Tom Moens[1], Janek Simon[4], Jeroen Van Wichelen[2], Håkan Westerberg[5], Klaus Wysujack[6], David Righton[7]

[1] Marine Biology Research Group, Ghent University, Krijgslaan 281, 9000 Ghent, Belgium

[2] Research Institute for Nature and Forest (INBO), Havenlaan 88, bus 73, 1000 Brussels, Belgium

[3] Flanders Marine Institute (VLIZ), Wandelaarkaai 7, 8400 Ostend, Belgium

[4] Institute of Inland Fisheries e.V. Potsdam Sacrow, Im Königswald 2, 14469 Potsdam, Germany

[5] Swedish University of Agricultural Sciences (SLU), Institute of Freshwater Research, 178 93 Drottningholm, Sweden

[6] Thünen Institute of Fisheries Ecology, Herwigstraße 31, 27572 Bremerhaven, Germany.

[7] Centre for Environment, Fisheries, and Aquaculture Science , Pakefield Road, Lowestoft, UK, NR33 0HT

* Corresponding author

Pieterjan Verhelst

Research Institute for Nature and Forest

Aquatic Management

Havenlaan 88, bus 73

1000 Brussels - Belgium

Tel.: +32 (0)499 38 72 87

pieterjan.verhelst@inbo.be

Supplemental information containing supplemental figures and tables.

Figures

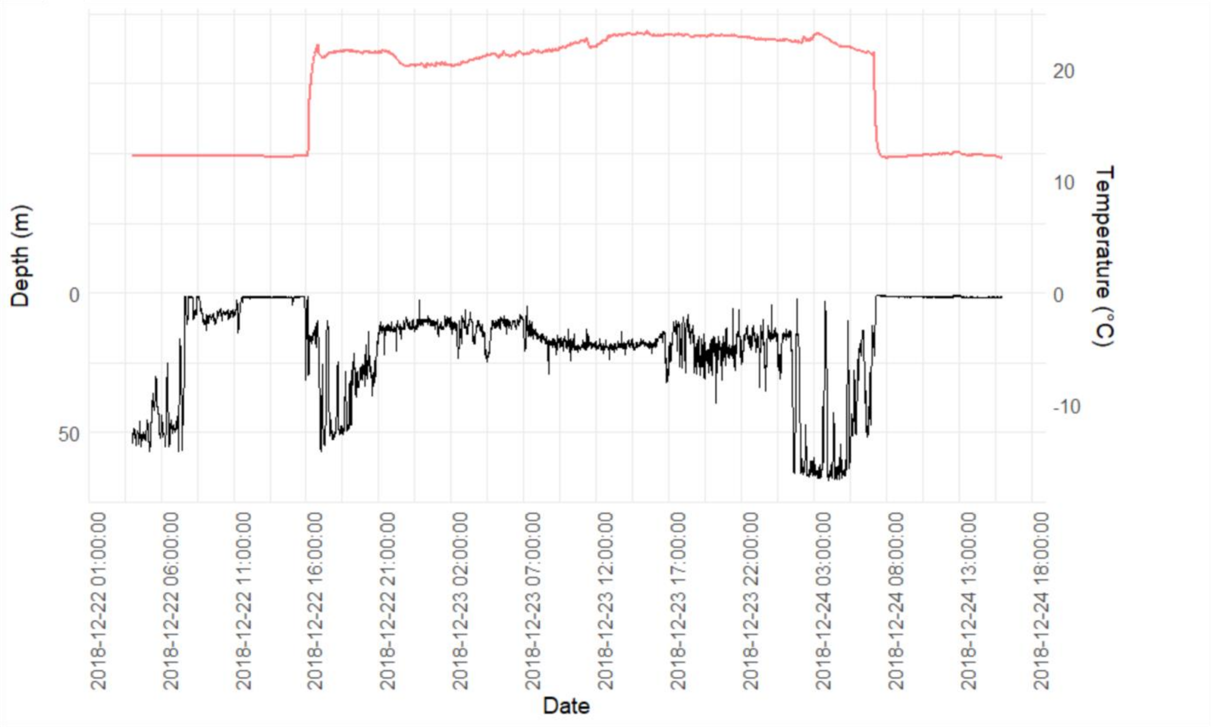

Figure S1. The temperature (red line, right y-axis) rose to ca. 23°C indicating the predation by an endothermic fish. The black line shows the vertical movement pattern (left y-axis). The time on the x-axis is in CET.

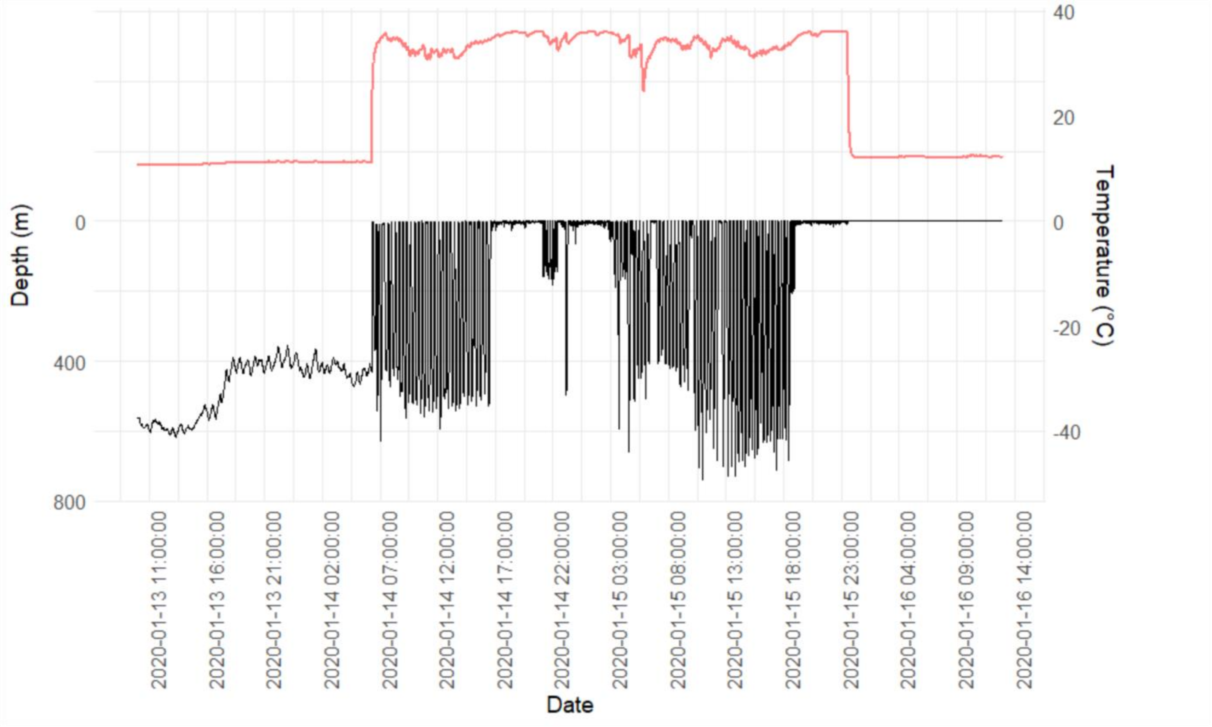

Figure S2. The temperature (red line, right y-axis) rose to ca. 35°C indicating the predation by a marine mammal. The predator was likely a pilot whale (*Globicephala sp.*) species as the animal dove frequently to depths > 400 m for 20 minutes (black line, left y-axis). The time on the x-axis is in CET.

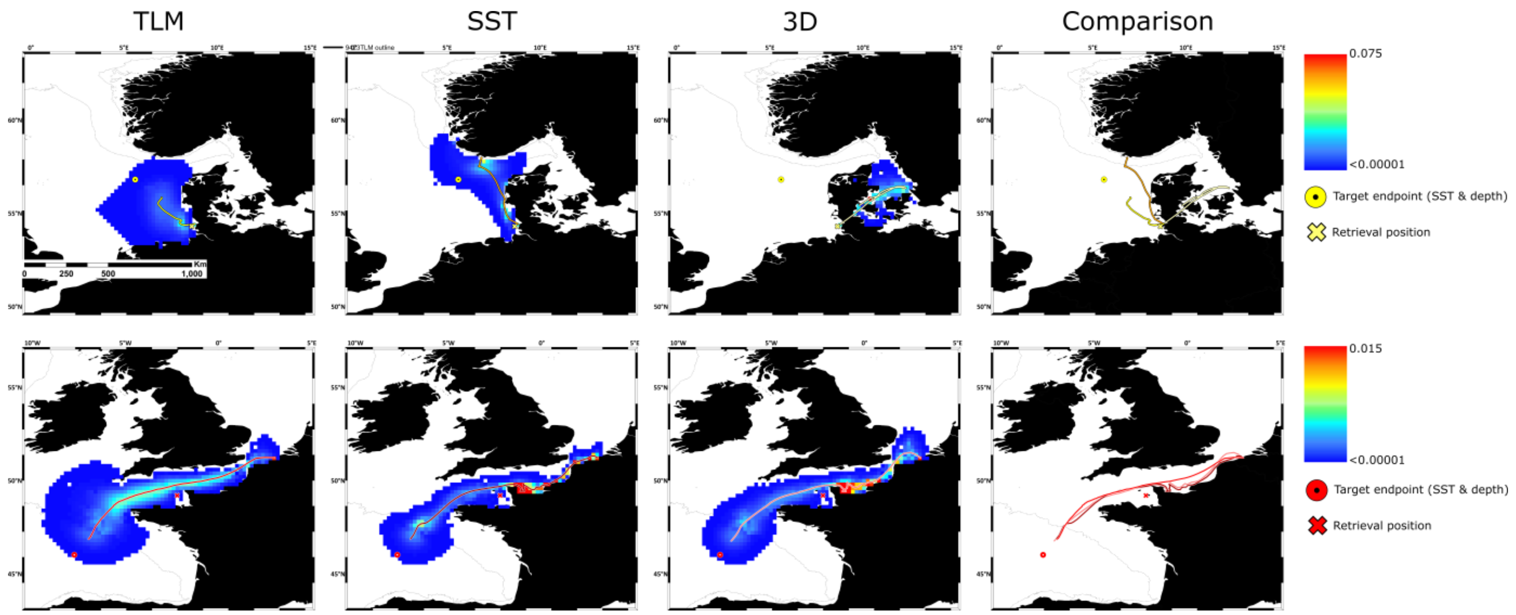

Figure S3. Outputs of the three different geolocation methods (TLM, SST and 3D) on example PDSTs from German (tag 9423, top row) and Belgian (tag 16031, bottom row) releases. Coloured areas show the utility distribution (UD) resulting from each geolocation, while lines show the most likely trajectory for each method.

Tables

Table S1. Table of the 320 tagged eels with the archival tag type (X-tag or PDST), tagging year, release location, total lenght (mm) and weight (g), the silver eel stage according to Durif et al. (2005), whether or not the tag was retrieved (y for yes and n for no) and the tag fate.

Tag ID	Archival tag type	Tagging year	Release location	Total Length (mm)	Weight (g)	Silver eel stage	Retrieved (y/n)	Tag fate
112061	X-tag	2011	River Eider, Germany	865	1528	FIV	y	premature release
112064	X-tag	2011	River Eider, Germany	925	1710	FIV	y	premature release
112063	X-tag	2011	River Eider, Germany	880	1357	FIV	y	premature release
112058	X-tag	2011	River Eider, Germany	910	1541	FIV	y	premature release
112059	X-tag	2011	River Eider, Germany	930	1579	FIV	n	
112060	X-tag	2011	River Eider, Germany	870	1220	FIV	n	
112062	X-tag	2011	River Eider, Germany	890	1484	FIV	n	
A09349	G5 DST	2012	River Eider, Germany	873	1182	FIV	y	premature release
A09350	G5 DST	2012	River Eider, Germany	867	1361	FIV	n	
A09351	G5 DST	2012	River Eider, Germany	904	1635	FIV	n	
A09353	G5 DST	2012	River Eider, Germany	844	1093	FIV	n	
A09354	G5 DST	2012	River Eider, Germany	733	771	FV	n	
A09355	G5 DST	2012	River Eider, Germany	722	765	FV	y	predated by unknown predator
A09356	G5 DST	2012	River Eider, Germany	720	645	FV	n	
A09357	G5 DST	2012	River Eider, Germany	785	799	FV	n	
A09358	G5 DST	2012	River Eider, Germany	738	904	FIV	y	premature release
A09359	G5 DST	2012	River Eider, Germany	758	833	FV	y	premature release
A09360	G5 DST	2012	River Eider, Germany	755	867	FV	n	

A09361	G5 DST	2012	River Eider, Germany	762	783	FV	n	
A09362	G5 DST	2012	River Eider, Germany	710	749	FV	y	premature release
A09363	G5 DST	2012	Elbe Estuary, Germany	754	708	FV	n	
A09364	G5 DST	2012	Elbe Estuary, Germany	726	665	FV	n	
A09365	G5 DST	2012	Elbe Estuary, Germany	826	1029	FIV	n	
A09366	G5 DST	2012	Elbe Estuary, Germany	757	802	FV	n	
A09367	G5 DST	2012	Elbe Estuary, Germany	716	761	FV	n	
A09368	G5 DST	2012	Elbe Estuary, Germany	808	1035	FIV	n	
A09369	G5 DST	2012	Elbe Estuary, Germany	857	1364	FIV	n	
A09370	G5 DST	2012	Elbe Estuary, Germany	745	717	FV	n	
A09371	G5 DST	2012	Elbe Estuary, Germany	824	1105	FIV	n	
A09372	G5 DST	2012	Elbe Estuary, Germany	704	649	FV	n	
A09373	G5 DST	2012	Elbe Estuary, Germany	742	816	FV	n	
A09374	G5 DST	2012	Elbe Estuary, Germany	762	770	FV	y	fished
A09375	G5 DST	2012	Elbe Estuary, Germany	778	812	FV	y	premature release
A09376	G5 DST	2012	River Eider, Germany	826	1060	FIV	n	
A09377	G5 DST	2012	River Eider, Germany	845	1197	FIV	y	programmed release
A09378	G5 DST	2012	River Eider, Germany	784	1103	FIV	n	
A09379	G5 DST	2012	Elbe Estuary, Germany	713	739	FV	n	
A09380	G5 DST	2012	Elbe Estuary, Germany	711	639	FV	n	
A09382	G5 DST	2012	Elbe Estuary, Germany	706	602	FV	n	
A09383	G5 DST	2012	Elbe Estuary, Germany	795	893	FV	n	

A09384	G5 DST	2012	Elbe Estuary, Germany	728	663	FV	n	
A09385	G5 DST	2012	Elbe Estuary, Germany	738	843	FV	n	
A09386	G5 DST	2012	Elbe Estuary, Germany	698	655	FV	y	premature release
A09387	G5 DST	2012	Elbe Estuary, Germany	751	731	FV	n	
A09388	G5 DST	2012	Elbe Estuary, Germany	743	712	FV	n	
A09389	G5 DST	2012	Elbe Estuary, Germany	668	639	FV	y	premature release
A09390	G5 DST	2012	Elbe Estuary, Germany	767	855	FV	n	
A09391	G5 DST	2012	Elbe Estuary, Germany	840	1105	FV	n	
A09392	G5 DST	2012	Elbe Estuary, Germany	806	1328	FIV	n	
A09393	G5 DST	2012	Elbe Estuary, Germany	714	701	FV	y	premature release
A09394	G5 DST	2012	Elbe Estuary, Germany	807	922	FV	n	
A09395	G5 DST	2012	Elbe Estuary, Germany	731	829	FV	n	
A09396	G5 DST	2012	Elbe Estuary, Germany	634	566	FV	n	
A09397	G5 DST	2012	Elbe Estuary, Germany	777	877	FV	n	
A09398	G5 DST	2012	Elbe Estuary, Germany	725	686	FV	n	
A09399	G5 DST	2012	Elbe Estuary, Germany	764	1156	FIV	n	
A09400	G5 DST	2012	Elbe Estuary, Germany	824	1094	FIV	n	
A09401	G5 DST	2012	Elbe Estuary, Germany	708	669	FV	n	
A09402	G5 DST	2012	Elbe Estuary, Germany	751	761	FV	n	
A09403	G5 DST	2012	River Eider, Germany	763	852	FV	n	
A09404	G5 DST	2012	River Eider, Germany	758	701	FV	n	
A09405	G5 DST	2012	River Eider, Germany	708	657	FV	n	

A09406	G5 DST	2012	River Eider, Germany	884	1540	FIV	n	
A09407	G5 DST	2012	River Eider, Germany	817	1180	FIV	n	
A09408	G5 DST	2012	River Eider, Germany	872	1542	FIV	n	
A09409	G5 DST	2012	River Eider, Germany	903	1890	FIV	n	
A09411	G5 DST	2012	River Eider, Germany	804	1190	FIV	y	premature release
A09413	G5 DST	2012	Elbe Estuary, Germany	710	789	FV	y	programmed release
A09414	G5 DST	2012	Elbe Estuary, Germany	754	929	FV	n	
A09416	G5 DST	2012	Elbe Estuary, Germany	754	783	FV	n	
A09418	G5 DST	2012	Elbe Estuary, Germany	724	749	FV	y	premature release
A09419	G5 DST	2012	Elbe Estuary, Germany	876	1114	FIV	n	
A09420	G5 DST	2012	Elbe Estuary, Germany	725	718	FV	n	
A09421	G5 DST	2012	Elbe Estuary, Germany	784	961	FIV	n	
A09422	G5 DST	2012	Elbe Estuary, Germany	767	898	FV	n	
A09423	G5 DST	2012	River Eider, Germany	693	657	FV	y	premature release
A09424	G5 DST	2012	Elbe Estuary, Germany	770	911	FV	y	premature release
A09425	G5 DST	2012	River Eider, Germany	708	877	FIV	n	
A09426	G5 DST	2012	River Eider, Germany	806	1287	FIV	n	
A09427	G5 DST	2012	River Eider, Germany	816	1156	FIV	n	
A09428	G5 DST	2012	River Eider, Germany	946	1919	FIV	n	
A09429	G5 DST	2012	River Eider, Germany	748	768	FV	n	
A15806	G5 DST	2018	Yser Estuary, Belgium	788	969	FV	y	premature release
A15802	G5 DST	2018	Yser Estuary, Belgium	712	759	FV	n	

A15801	G5 DST	2018	Yser Estuary, Belgium	667	823	FV	n	
A15797	G5 DST	2018	Yser Estuary, Belgium	724	740	FV	n	
A15799	G5 DST	2018	Yser Estuary, Belgium	800	963	FV	y	premature release
A15803	G5 DST	2018	Yser Estuary, Belgium	888	1190	FIV	n	
A15805	G5 DST	2018	Yser Estuary, Belgium	928	1394	FIV	y	premature release
A15804	G5 DST	2018	Yser Estuary, Belgium	842	1191	FIV	y	premature release
A15807	G5 DST	2018	Yser Estuary, Belgium	780	934	FIV	y	premature release
A15730	G5 DST	2018	Yser Estuary, Belgium	770	1025	FIV	y	premature release
A15739	G5 DST	2018	Yser Estuary, Belgium	825	983	FV	n	
A15740	G5 DST	2018	Yser Estuary, Belgium	806	1023	FIV	n	
A15733	G5 DST	2018	Yser Estuary, Belgium	827	1348	FIV	y	premature release
A15729	G5 DST	2018	Yser Estuary, Belgium	802	970	FV	y	premature release
A15731	G5 DST	2018	Yser Estuary, Belgium	858	1162	FV	n	
A15756	G5 DST	2018	Yser Estuary, Belgium	782	925	FV	n	
A15743	G5 DST	2018	Yser Estuary, Belgium	835	1092	FIV	n	
A15735	G5 DST	2018	Yser Estuary, Belgium	884	1275	FIV	n	
A15757	G5 DST	2018	Yser Estuary, Belgium	980	1704	FIV	y	premature release
A15748	G5 DST	2018	Yser Estuary, Belgium	855	1193	FIV	n	
A15752	G5 DST	2018	Yser Estuary, Belgium	859	1158	FV	y	premature release
A15736	G5 DST	2018	Yser Estuary, Belgium	816	1093	FIV	y	premature release
A15750	G5 DST	2018	Yser Estuary, Belgium	788	944	FV	n	
A15732	G5 DST	2018	Yser Estuary, Belgium	782	786	FV	n	

A15700	G5 DST	2018	Yser Estuary, Belgium	817	1051	FIV	y	premature release
A15741	G5 DST	2018	Yser Estuary, Belgium	742	794	FV	n	
A15755	G5 DST	2018	Yser Estuary, Belgium	842	1272	FIV	n	
A15746	G5 DST	2018	Yser Estuary, Belgium	813	1211	FIV	y	premature release
A15760	G5 DST	2018	Yser Estuary, Belgium	831	1403	FIV	y	premature release
A15763	G5 DST	2018	Yser Estuary, Belgium	790	1156	FIV	n	
A15747	G5 DST	2018	Yser Estuary, Belgium	989	1839	FIV	y	premature release
A15699	G5 DST	2018	Yser Estuary, Belgium	758	925	FIII	n	
A15753	G5 DST	2018	Yser Estuary, Belgium	830	1080	FIV	n	
A15711	G5 DST	2018	Yser Estuary, Belgium	809	1044	FIV	n	
A15708	G5 DST	2018	Yser Estuary, Belgium	756	809	FV	n	
A15745	G5 DST	2018	Yser Estuary, Belgium	720	908	FV	n	
A15761	G5 DST	2018	Yser Estuary, Belgium	802	982	FV	n	
A15758	G5 DST	2018	Yser Estuary, Belgium	795	1039	FV	n	
A15751	G5 DST	2018	Yser Estuary, Belgium	875	1169	FIV	n	
A15749	G5 DST	2018	Yser Estuary, Belgium	785	933	FV	n	
A15701	G5 DST	2018	Yser Estuary, Belgium	782	874	FV	n	
A15762	G5 DST	2018	Yser Estuary, Belgium	815	1001	FV	n	
A15720	G5 DST	2018	Yser Estuary, Belgium	772	888	FIV	n	
A15716	G5 DST	2018	Yser Estuary, Belgium	742	788	FV	y	premature release
A15713	G5 DST	2018	Yser Estuary, Belgium	915	1519	FIV	n	
A15719	G5 DST	2018	Yser Estuary, Belgium	780	1042	FIV	n	

A15707	G5 DST	2018	Yser Estuary, Belgium	870	1366	FIV	n	
A15710	G5 DST	2018	Yser Estuary, Belgium	775	884	FV	n	
A15714	G5 DST	2018	Yser Estuary, Belgium	746	917	FIV	y	predated by endothermic fish
A15715	G5 DST	2018	Yser Estuary, Belgium	763	807	FV	n	
A15724	G5 DST	2018	Yser Estuary, Belgium	814	1048	FV	n	
A15798	G5 DST	2018	Yser Estuary, Belgium	806	1027	FV	n	
A15717	G5 DST	2018	Yser Estuary, Belgium	827	1088	FV	n	
A15722	G5 DST	2018	Yser Estuary, Belgium	758	1003	FIV	n	
A15721	G5 DST	2018	Yser Estuary, Belgium	750	787	FV	n	
A15728	G5 DST	2018	Yser Estuary, Belgium	847	1224	FIV	n	
A15723	G5 DST	2018	Yser Estuary, Belgium	695	731	FV	n	
A15727	G5 DST	2018	Yser Estuary, Belgium	823	1164	FIV	n	
A15725	G5 DST	2018	Yser Estuary, Belgium	773	961	FV	n	
A15726	G5 DST	2018	Yser Estuary, Belgium	777	817	FV	n	
A15767	G5 DST	2018	Yser Estuary, Belgium	760	953	FV	n	
A15776	G5 DST	2018	Yser Estuary, Belgium	794	871	FV	n	
A15770	G5 DST	2018	Yser Estuary, Belgium	800	888	FV	n	
A15765	G5 DST	2018	Yser Estuary, Belgium	826	1129	FIV	n	
A15768	G5 DST	2018	Yser Estuary, Belgium	774	1045	FV	n	
A15766	G5 DST	2018	Yser Estuary, Belgium	822	1076	FV	n	
A15772	G5 DST	2018	Yser Estuary, Belgium	800	908	FV	y	premature release
A15702	G5 DST	2018	Yser Estuary, Belgium	752	948	FIV	n	

A15771	G5 DST	2018	Yser Estuary, Belgium	1025	2086	FIV	n	
A15703	G5 DST	2018	Yser Estuary, Belgium	822	1125	FIV	n	
A15764	G5 DST	2018	Yser Estuary, Belgium	823	976	FIV	n	
A15705	G5 DST	2018	Yser Estuary, Belgium	794	928	FV	n	
A15718	G5 DST	2018	Yser Estuary, Belgium	751	862	FV	y	premature release
A16031	G5 DST	2018	Yser Estuary, Belgium	817	991	FV	y	premature release
A15706	G5 DST	2018	Yser Estuary, Belgium	738	811	FIII	y	premature release
A15982	G5 DST	2018	Yser Estuary, Belgium	774	865	FV	y	premature release
A15796	G5 DST	2018	Yser Estuary, Belgium	795	931	FV	n	
A15769	G5 DST	2018	Yser Estuary, Belgium	763	885	FV	y	premature release
A15981	G5 DST	2018	Yser Estuary, Belgium	785	1136	FIV	y	premature release
A15704	G5 DST	2018	Yser Estuary, Belgium	732	876	FIV	n	
A15785	G5 DST	2018	Yser Estuary, Belgium	825	1273	FIV	y	premature release
A15777	G5 DST	2018	Yser Estuary, Belgium	770	896	FIV	y	predated by unknown predator
A15781	G5 DST	2018	Yser Estuary, Belgium	804	980	FV	n	
A15778	G5 DST	2018	Yser Estuary, Belgium	824	1091	FIV	n	
A15784	G5 DST	2018	Yser Estuary, Belgium	837	1174	FIV	n	
A15779	G5 DST	2018	Yser Estuary, Belgium	833	1150	FV	y	premature release
A15773	G5 DST	2018	Yser Estuary, Belgium	852	1106	FIV	n	
A15774	G5 DST	2018	Yser Estuary, Belgium	887	1473	FIV	n	
A15780	G5 DST	2018	Yser Estuary, Belgium	774	1079	FIV	y	premature release
A15775	G5 DST	2018	Yser Estuary, Belgium	784	1010	FIII	n	

A15782	G5 DST	2018	Yser Estuary, Belgium	870	1314	FIV	n	
A15786	G5 DST	2018	Yser Estuary, Belgium	733	779	FV	n	
A15789	G5 DST	2018	Yser Estuary, Belgium	713	808	FIV	y	premature release
A15788	G5 DST	2018	Yser Estuary, Belgium	820	1027	FIV	n	
A15787	G5 DST	2018	Yser Estuary, Belgium	741	764	FIII	n	
A15795	G5 DST	2018	Yser Estuary, Belgium	810	1095	FIV	n	
A15794	G5 DST	2018	Yser Estuary, Belgium	717	1017	FIV	n	
A15790	G5 DST	2018	Yser Estuary, Belgium	813	1126	FIV	n	
A15792	G5 DST	2018	Yser Estuary, Belgium	829	1181	FIV	n	
A15799	G5 DST	2018	Yser Estuary, Belgium	842	1052	FIV	n	
A15733	G5 DST	2018	Yser Estuary, Belgium	879	1717	FIV	n	
A15757	G5 DST	2018	Yser Estuary, Belgium	893	1443	FIV	n	
A17443	G5 DST	2019	Yser Estuary, Belgium	821	1046	FIV	y	premature release
A17447	G5 DST	2019	Yser Estuary, Belgium	892	1277	FIV	y	premature release
A17449	G5 DST	2019	Yser Estuary, Belgium	801	1154	FIV	y	premature release
A17482	G5 DST	2019	Yser Estuary, Belgium	827	1065	FIV	n	
A17451	G5 DST	2019	Yser Estuary, Belgium	823	1116	FIV	y	premature release
A17492	G5 DST	2019	Yser Estuary, Belgium	805	1053	FIV	y	premature release
A17507	G5 DST	2019	Yser Estuary, Belgium	785	985	FIV	n	
A17500	G5 DST	2019	Yser Estuary, Belgium	793	1084	FIV	n	
A17502	G5 DST	2019	Yser Estuary, Belgium	800	1174	FIV	n	
A17501	G5 DST	2019	Yser Estuary, Belgium	787	1028	FIV	n	

A17496	G5 DST	2019	Yser Estuary, Belgium	863	1248	FIV	n	
A17497	G5 DST	2019	Yser Estuary, Belgium	793	906	FV	n	
A17498	G5 DST	2019	Yser Estuary, Belgium	865	1288	FIV	n	
A17499	G5 DST	2019	Yser Estuary, Belgium	830	1044	FIV	y	premature release
A17509	G5 DST	2019	Yser Estuary, Belgium	723	901	FV	n	
A17503	G5 DST	2019	Yser Estuary, Belgium	798	1112	FIV	y	premature release
A17510	G5 DST	2019	Yser Estuary, Belgium	775	996	FIV	y	premature release
A17528	G5 DST	2019	Yser Estuary, Belgium	845	1216	FIV	y	premature release
A17534	G5 DST	2019	Yser Estuary, Belgium	916	1758	FIV	y	premature release
A17526	G5 DST	2019	Yser Estuary, Belgium	887	1694	FIV	y	premature release
A17520	G5 DST	2019	Yser Estuary, Belgium	811	1052	FV	n	
A17511	G5 DST	2019	Yser Estuary, Belgium	855	1003	FIV	n	
A17508	G5 DST	2019	Yser Estuary, Belgium	805	1148	FIV	y	premature release
A17515	G5 DST	2019	Yser Estuary, Belgium	730	824	FV	n	
A17514	G5 DST	2019	Yser Estuary, Belgium	771	978	FIV	n	
A17506	G5 DST	2019	Yser Estuary, Belgium	894	1417	FIV	n	
A17513	G5 DST	2019	Yser Estuary, Belgium	831	1274	FIV	y	premature release
A17512	G5 DST	2019	Yser Estuary, Belgium	873	1340	FIV	n	
A17519	G5 DST	2019	Yser Estuary, Belgium	877	1347	FIV	n	
A17516	G5 DST	2019	Yser Estuary, Belgium	805	1267	FIV	n	
A17518	G5 DST	2019	Yser Estuary, Belgium	880	1286	FIV	y	premature release
A17517	G5 DST	2019	Yser Estuary, Belgium	786	909	FV	n	

A17505	G5 DST	2019	Yser Estuary, Belgium	845	1238	FIV	n	
A17524	G5 DST	2019	Yser Estuary, Belgium	778	841	FV	n	
A17522	G5 DST	2019	Yser Estuary, Belgium	811	1123	FIV	y	premature release
A17521	G5 DST	2019	Yser Estuary, Belgium	753	901	FV	y	predated by unknown predator
A17504	G5 DST	2019	Yser Estuary, Belgium	779	962	FV	n	
A17540	G5 DST	2019	Yser Estuary, Belgium	811	1137	FIV	n	
A17541	G5 DST	2019	Yser Estuary, Belgium	829	1324	FIV	n	
A17527	G5 DST	2019	Yser Estuary, Belgium	838	1133	FIV	n	
A17530	G5 DST	2019	Yser Estuary, Belgium	767	823	FV	n	
A17536	G5 DST	2019	Yser Estuary, Belgium	820	1231	FIV	y	premature release
A17535	G5 DST	2019	Yser Estuary, Belgium	877	1518	FIV	y	predated by mammal
A17525	G5 DST	2019	Yser Estuary, Belgium	979	805	FIII	y	premature release
A17533	G5 DST	2019	Yser Estuary, Belgium	809	1192	FIV	y	probably predated
A17532	G5 DST	2019	Yser Estuary, Belgium	730	911	FIV	y	premature release
A17531	G5 DST	2019	Yser Estuary, Belgium	834	1090	FIV	n	
A17538	G5 DST	2019	Yser Estuary, Belgium	779	986	FIV	y	premature release
A17539	G5 DST	2019	Yser Estuary, Belgium	956	1991	FIV	n	
A17537	G5 DST	2019	Yser Estuary, Belgium	856	1501	FIV	y	premature release
A15729	G5 DST	2019	Yser Estuary, Belgium	908	1514	FIV	n	
A15785	G5 DST	2019	Yser Estuary, Belgium	1053	2067	FIV	n	
A15806	G5 DST	2019	Yser Estuary, Belgium	862	1446	FIV	n	
A15736	G5 DST	2019	Yser Estuary, Belgium	968	1748	FIV	n	

A15746	G5 DST	2019	Yser Estuary, Belgium	914	1555	FIV	y	premature release
A15780	G5 DST	2019	Yser Estuary, Belgium	844	1240	FIV	n	
A15789	G5 DST	2019	Yser Estuary, Belgium	807	1254	FIV	y	premature release
A15714	G5 DST	2019	Yser Estuary, Belgium	891	1719	FIV	n	
A15700	G5 DST	2019	Yser Estuary, Belgium	912	1983	FIV	y	premature release
A15730	G5 DST	2019	Yser Estuary, Belgium	845	1346	FIV	y	premature release
A17659	G5 DST	2020	Yser Estuary, Belgium	822	1165	FIV	n	
A17650	G5 DST	2020	Yser Estuary, Belgium	756	1220	FIV	n	
A17649	G5 DST	2020	Yser Estuary, Belgium	784	1133	FIV	n	
A17665	G5 DST	2020	Yser Estuary, Belgium	801	1061	FIV	n	
A17658	G5 DST	2020	Yser Estuary, Belgium	890	1322	FIV	y	premature release
A17666	G5 DST	2020	Yser Estuary, Belgium	895	1302	FIV	n	
A17653	G5 DST	2020	Yser Estuary, Belgium	786	978	FIV	y	premature release
A17661	G5 DST	2020	Yser Estuary, Belgium	782	923	FV	n	
A17667	G5 DST	2020	Yser Estuary, Belgium	814	1110	FIV	n	
A17656	G5 DST	2020	Yser Estuary, Belgium	782	1020	FIV	n	
A17668	G5 DST	2020	Yser Estuary, Belgium	976	1862	FIV	y	predated by mammal
A17664	G5 DST	2020	Yser Estuary, Belgium	791	1033	FIV	n	
A17662	G5 DST	2020	Yser Estuary, Belgium	847	1184	FIV	n	
A17645	G5 DST	2020	Yser Estuary, Belgium	825	1137	FIV	n	
A17646	G5 DST	2020	Yser Estuary, Belgium	745	942	FIV	y	premature release
A17652	G5 DST	2020	Yser Estuary, Belgium	859	1252	FIV	y	predated by endothermic fish

A17648	G5 DST	2020	Yser Estuary, Belgium	830	1505	FIV	y	premature release
A17644	G5 DST	2020	Yser Estuary, Belgium	817	1176	FIV	n	
A17663	G5 DST	2020	Yser Estuary, Belgium	761	853	FV	y	premature release
A17660	G5 DST	2020	Yser Estuary, Belgium	856	1291	FIV	y	premature release
A17627	G5 DST	2020	Yser Estuary, Belgium	738	892	FIV	n	
A17642	G5 DST	2020	Yser Estuary, Belgium	732	835	FV	y	premature release
A17651	G5 DST	2020	Yser Estuary, Belgium	747	881	FV	n	
A17654	G5 DST	2020	Yser Estuary, Belgium	758	966	FIV	n	
A17631	G5 DST	2020	Yser Estuary, Belgium	952	1658	FIV	n	
A17634	G5 DST	2020	Yser Estuary, Belgium	863	1098	FIV	y	premature release
A17629	G5 DST	2020	Yser Estuary, Belgium	773	934	FIV	n	
A17633	G5 DST	2020	Yser Estuary, Belgium	811	984	FIV	n	
A17636	G5 DST	2020	Yser Estuary, Belgium	747	903	FIV	n	
A17639	G5 DST	2020	Yser Estuary, Belgium	869	1450	FIV	n	
A17544	G5 DST	2020	Yser Estuary, Belgium	908	1482	FIV	n	
A17548	G5 DST	2020	Yser Estuary, Belgium	908	838	FV	n	
A17626	G5 DST	2020	Yser Estuary, Belgium	824	1034	FIV	n	
A17647	G5 DST	2020	Yser Estuary, Belgium	780	876	FV	n	
A17545	G5 DST	2020	Yser Estuary, Belgium	775	1011	FIV	n	
A17623	G5 DST	2020	Yser Estuary, Belgium	786	1018	FIV	n	
A17655	G5 DST	2020	Yser Estuary, Belgium	837	1398	FIV	n	
A17640	G5 DST	2020	Yser Estuary, Belgium	1001	1893	FIV	n	

A17635	G5 DST	2020	Yser Estuary, Belgium	830	1280	FIV	y	predated by mammal
A17638	G5 DST	2020	Yser Estuary, Belgium	755	1143	FIV	y	premature release
A17637	G5 DST	2020	Yser Estuary, Belgium	876	1462	FIV	n	
A17630	G5 DST	2020	Yser Estuary, Belgium	852	1339	FIV	n	
A17632	G5 DST	2020	Yser Estuary, Belgium	780	1023	FIV	n	
A17628	G5 DST	2020	Yser Estuary, Belgium	796	1138	FIV	n	
A17625	G5 DST	2020	Yser Estuary, Belgium	944	1610	FIV	n	
A17624	G5 DST	2020	Yser Estuary, Belgium	870	1369	FIV	n	
A17547	G5 DST	2020	Yser Estuary, Belgium	850	1023	FV	y	predated by endothermic fish
A17546	G5 DST	2020	Yser Estuary, Belgium	788	1102	FIV	n	
A17543	G5 DST	2020	Yser Estuary, Belgium	806	1051	FIV	n	
A17542	G5 DST	2020	Yser Estuary, Belgium	862	1267	FIV	n	
A15783	G5 DST	2020	Yser Estuary, Belgium	802	1086	FIV	y	premature release
A17440	G5 DST	2020	Yser Estuary, Belgium	760	972	FIV	n	
A17521	G5 DST	2020	Yser Estuary, Belgium	757	965	FIV	n	
A17510	G5 DST	2020	Yser Estuary, Belgium	777	1067	FIV	n	
A17525	G5 DST	2020	Yser Estuary, Belgium	837	1203	FIV	y	premature release
A17538	G5 DST	2020	Yser Estuary, Belgium	836	1035	FV	n	
A17492	G5 DST	2020	Yser Estuary, Belgium	855	1281	FIV	y	premature release
A17508	G5 DST	2020	Yser Estuary, Belgium	832	1238	FIV	n	
A17537	G5 DST	2020	Yser Estuary, Belgium	828	1109	FV	n	
A17536	G5 DST	2020	Yser Estuary, Belgium	861	1306	FIV	n	

A17522	G5 DST	2020	Yser Estuary, Belgium	913	1524	FIV	n	
A17518	G5 DST	2020	Yser Estuary, Belgium	951	1639	FIV	y	premature release
A17526	G5 DST	2020	Yser Estuary, Belgium	765	1004	FV	n	
A17499	G5 DST	2020	Yser Estuary, Belgium	811	1328	FIV	y	premature release
A17513	G5 DST	2020	Yser Estuary, Belgium	810	1064	FIV	y	predated by endothermic fish
A17528	G5 DST	2020	Yser Estuary, Belgium	892	1467	FIV	n	
A17453	G5 DST	2020	Yser Estuary, Belgium	786	1072	FIV	n	
A17487	G5 DST	2020	Yser Estuary, Belgium	875	1461	FIV	y	premature release
A17443	G5 DST	2020	Yser Estuary, Belgium	816	1159	FIV	n	
A17466	G5 DST	2020	Yser Estuary, Belgium	836	1233	FIV	n	
A17532	G5 DST	2020	Yser Estuary, Belgium	792	1058	FIV	n	
A17488	G5 DST	2020	Yser Estuary, Belgium	824	1296	FIV	n	
A17447	G5 DST	2020	Yser Estuary, Belgium	768	914	FV	n	
A17449	G5 DST	2020	Yser Estuary, Belgium	786	1022	FIV	n	
A17461	G5 DST	2020	Yser Estuary, Belgium	836	1212	FIV	n	
A17480	G5 DST	2020	Yser Estuary, Belgium	967	1212	FIV	n	

Table S2. Table with the 54 archival tags that were included in the migration route reconstruction and migration speed analysis. For migration direction, N stands for northward and S for southward.

**Tag ID**	**Tag type**	**Release location**	**Release date**	**Release month**	**Total distance (km)**	**Total tracking days**	**Overall migration speed (km/day)**	**Migration direction**	**Total length (mm)**	**Weight (g)**
112058	PSAT	River Eider, Germany	23/12/2011	December	243	24	10	N	910	1541
112061	PSAT	River Eider, Germany	23/12/2011	December	759	68	11	N	925	1710
112064	PSAT	River Eider, Germany	23/12/2011	December	199	26	8	N	865	1528
A15700	PDST	Yser Estuary, Belgium	11/11/18	November	117	4	29	SW	817	1051
A15700_2	PDST	Yser Estuary, Belgium	17/12/2019	December	806	51	16	SW	912	1983
A15706	PDST	Yser Estuary, Belgium	9/12/2018	December	511	60	9	SW	738	811
A15714	PDST	Yser Estuary, Belgium	4/12/2018	December	402	16	25	SW	746	917
A15730	PDST	Yser Estuary, Belgium	31/10/18	November	226	8	28	SW	770	1025
A15730_2	PDST	Yser Estuary, Belgium	17/12/2019	December	783	39	20	SW	845	1346
A15757	PDST	Yser Estuary, Belgium	31/10/18	October	200	5	40	SW	980	1704
A15777	PDST	Yser Estuary, Belgium	9/12/2018	December	824	37	22	SW	770	896
A15789	PDST	Yser Estuary, Belgium	17/12/2019	December	1145	59	19	SW	807	1254
A15805	PDST	Yser Estuary, Belgium	31/10/18	November	229	7	33	N	928	1394
A15981	PDST	Yser Estuary,	9/12/2018	December	215	13	17	N	785	1136

		Belgium								
A16031	PDST	Yser Estuary, Belgium	9/12/2018	December	943	68	14	SW	817	991
A17443	PDST	Yser Estuary, Belgium	2/11/2019	November	546	26	21	SW	821	1046
A17487	PDST	Yser Estuary, Belgium	25/12/2020	December	771	25	31	SW	875	1460.6
A17492	PDST	Yser Estuary, Belgium	29/11/2019	November	800	29	28	SW	805	1053
A17492_2	PDST	Yser Estuary, Belgium	24/12/2020	December	455	37	12	N	855	1281.3
A17499	PDST	Yser Estuary, Belgium	29/11/2019	November	276	19	15	N	830	1044
A17499_2	PDST	Yser Estuary, Belgium	25/12/2020	December	257	38	7	SW	811	1328.3
A17508	PDST	Yser Estuary, Belgium	13/12/2019	December	809	34	24	SW	805	1148
A17510	PDST	Yser Estuary, Belgium	5/12/2019	December	861	40	22	SW	775	996
A17513	PDST	Yser Estuary, Belgium	14/12/2019	December	623	15	42	SW	831	1274
A17513_2	PDST	Yser Estuary, Belgium	25/12/2020	December	239	12	20	SW	810	1064.1
A17518_2	PDST	Yser Estuary, Belgium	25/12/2020	December	774	26	30	SW	951	1638.9
A17521	PDST	Yser Estuary, Belgium	14/12/2019	December	763	30	25	SW	753	901
A17522	PDST	Yser Estuary, Belgium	14/12/2019	December	637	30	21	SW	811	1123
A17525_2	PDST	Yser Estuary, Belgium	24/12/2020	December	359	26	14	N	837	1202.6
A17526	PDST	Yser Estuary,	10/12/2019	December	768	26	30	SW	887	1694

		Belgium								
A17534	PDST	Yser Estuary, Belgium	10/12/2019	December	778	25	31	SW	916	1758
A17535	PDST	Yser Estuary, Belgium	14/12/2019	December	876	29	30	SW	877	1518
A17536	PDST	Yser Estuary, Belgium	14/12/2019	December	816	27	30	SW	820	1231
A17537	PDST	Yser Estuary, Belgium	17/12/2019	December	944	30	31	SW	856	1501
A17538	PDST	Yser Estuary, Belgium	14/12/2019	December	768	30	26	SW	779	986
A17547	PDST	Yser Estuary, Belgium	24/12/2020	December	524	15	35	SW	850	1023.3
A17634	PDST	Yser Estuary, Belgium	22/12/2020	December	348	12	29	SW	863	1098.1
A17635	PDST	Yser Estuary, Belgium	24/12/2020	December	764	27	28	SW	830	1279.9
A17638	PDST	Yser Estuary, Belgium	24/12/2020	December	593	14	42	SW	755	1143
A17642	PDST	Yser Estuary, Belgium	15/12/2020	December	543	12	45	SW	732	834.9
A17646	PDST	Yser Estuary, Belgium	18/11/2020	November	315	15	21	SW	745	942.3
A17648	PDST	Yser Estuary, Belgium	4/12/2020	December	1013	40	25	SW	830	1504.8
A17653	PDST	Yser Estuary, Belgium	15/11/2020	November	661	20	33	SW	786	978.4
A17658	PDST	Yser Estuary, Belgium	15/11/2020	November	265	11	24	SW	890	1321.8
A17663	PDST	Yser Estuary, Belgium	6/12/2020	December	829	36	23	SW	761	852.6
A09349	PDST	River Eider,	26/09/12	September	273	37	7	N	873	1182

		Germany								
A09358	PDST	River Eider, Germany	27/09/12	September	249	30	8	N	738	904
A09359	PDST	River Eider, Germany	27/09/12	September	223	20	11	N	758	833
A09374	PDST	Elbe Estuary, Germany	14/11/12	November	710	33	22	SW	762	770
A09377	PDST	River Eider, Germany	10/10/12	October	345	48	7	SW	845	1197
A09393	PDST	Elbe Estuary, Germany	14/11/12	November	510	31	16	N	714	701
A09411	PDST	River Eider, Germany	10/10/12	October	196	15	13	N	804	1190
A09423	PDST	River Eider, Germany	26/09/12	September	276	33	8	N	693	657
A09424	PDST	Elbe Estuary, Germany	22/11/12	November	333	21	16	N	770	911

Table S3. Specifications of the archival tags used in this study with their sensor settings.

Tag type	Dimensions (length x diameter) (mm)	Weight in air (g)	Apparent weight in seawater (g)	Sensors	Resolution	Sampling period (s)	Programmed pop-off period
G5 PDST (Cefas)	61 x 30	32	-0.1	temperature	0.031°C	10	6 or 12 months
				100 bar pressure	0.3 m	2	
G5 PDST (Cefas)	93 x 23	31	-0.5	temperature	0.031°C	120	50, 70 or 90 days
				50 or 100 bar pressure	0.15 or 0.3 m, respectively	10	
X-Tag (Microwave Telemetry Inc.)	120 x 32 (excluding the 185 mm antenna)	46	-3.8	temperature	0.16 to 0.23°C	120	12 months
				pressure	0.34–5.4 m	120	
